# Soluble CD83 Regulates Dendritic Cell–T Cell Immunological Synapse Formation by Disrupting Rab1a-Mediated F-Actin Rearrangement

**DOI:** 10.3389/fcell.2020.605713

**Published:** 2021-01-22

**Authors:** Wei Lin, Shuping Zhou, Meng Feng, Yong Yu, Qinghong Su, Xiaofan Li

**Affiliations:** Institute of Basic Medicine, Shandong Provincial Hospital Affiliated to Shandong First Medical University, Shandong First Medical University & Shandong Academy of Medical Science, Jinan, China

**Keywords:** sCD83, DC-T contact, Rab1A, immunological synapse, autoimmune uveitis

## Abstract

Dendritic cell–T cell (DC-T) contacts play an important role in T cell activation, clone generation, and development. Regulating the cytoskeletal protein rearrangement of DCs can modulate DC-T contact and affect T cell activation. However, inhibitory factors on cytoskeletal regulation in DCs remain poorly known. We showed that a soluble form of CD83 (sCD83) inhibited T cell activation by decreasing DC-T contact and synapse formation between DC and T cells. This negative effect of sCD83 on DCs was mediated by disruption of F-actin rearrangements, leading to alter expression and localization of major histocompatibility complex class II (MHC-II) and immunological synapse formation between DC and T cells. Furthermore, sCD83 was found to decrease GTP-binding activity of Rab1a, which further decreased colocalization and expression of LRRK2 and F-actin rearrangements in DCs, leading to the loss of MHC-II at DC-T synapses and reduced DC-T synapse formation. Further, sCD83-treated DCs alleviated symptoms of experimental autoimmune uveitis in mice and decreased the number of T cells in the eyes and lymph nodes of these animals. Our findings demonstrate a novel signaling pathway of sCD83 on regulating DC-T contact, which may be harnessed to develop new immunosuppressive therapeutics for autoimmune disease.

## Introduction

Dendritic cells (DCs) play a crucial regulatory role in autoimmune disease and regulate the immune response by governing T cell activation or development. Immunological synapses (ISs) formed between DCs and T cells (DC-T) support direct communication between cells and are a key factor for T cell clone generation and development (Dustin and Shaw, 1999; Grakoui et al., 1999; Davis and Dustin, 2004). IS of DC-T contacts is a multimolecular assembly of receptors and adhesion molecules that act as a platform for cell activation and cell–cell communication (Dustin and Shaw, 1999; Grakoui et al., 1999; Davis and Dustin, 2004), and it is formed by cytoskeletal proteins around one or more major histocompatibility complex class II–peptide–T cell receptor (MHC-II–peptide–TCR) cluster. Regulating the IS of DC-T would influence T cell activation directly. Cytoskeletal rearrangement is one of the most important factors in altering DC-T contacts (Al-Alwan et al., 2001; Comrie et al., 2015). F-actin provides essential support for the platform on the DC side of the synapse (Al-Alwan et al., 2001) and could regulate DC-T contact and T cell activation (Lin et al., 2015; Chen et al., 2017). Furthermore, F-actin acts as a scaffold to sustain signaling pathway molecules and controls the spatial and temporal distribution of Ca^2+^ sources and sinks to influence the activation and maturation of DCs toward DC-T contact and T cell activation (Nolz et al., 2006; Quintana et al., 2006, 2007). Therefore, inhibition of the cytoskeleton of DCs would influence DC-T synapse formation and the aggregation of immune-stimulating molecules between DC–T cells, which as a result would further decrease DC-T contact and T cell activation. Superfamily small GTPases (e.g., Rho and Ras) are believed to control cytoskeletal function through the regulation of effector kinases. Recently, Rab1a is found to control the actin cytoskeleton by regulating Roco2 kinase activity (Kicka et al., 2011), although its major function is related to vesicle trafficking and autophagy (Ali et al., 2004; Webster et al., 2016). It indicates that Rab1a may participate to the DC-T IS formation. However, the regulatory mechanism of DC-T contact formation is still not very clear.

Soluble CD83 (sCD83) is an immunosuppressive mediator involved in the pathogenesis of immune-related diseases in humans such as multiple sclerosis and animal models of experimental autoimmune encephalomyelitis (Pashine et al., 2008), experimental autoimmune uveitis (EAU) (Lin et al., 2018), systemic lupus erythematosus (Starke et al., 2013), and transplant rejection (Xu et al., 2007; Lan et al., 2010). These studies investigated the effects of sCD83 inhibition on DC regulation by activation of interleukin 10 (IL-10) and indoleamine 2,3-dioxygenase, which further suppresses T cell activation (Lan et al., 2010; Lin et al., 2018). In the same study, we also showed that sCD83 inhibits calcium release in DCs to suppress T cell activation (Lin et al., 2018). A different study reported that sCD83 completely changes the cytoskeleton of mature DCs, altering cells to become rounded and having either short or truncated cytoplasmic veils or no veils at all (Kotzor et al., 2004). They also found that sCD83-treated cells were completely inhibited in their ability to stimulate T cells (Kotzor et al., 2004). Nevertheless, the mechanism underlying the effect of sCD83 on DCs remains unclear, and how sCD83-mediated regulation of the DC cytoskeleton inhibits DC-T contact and T cell activation is still unknown.

Autoimmune uveitis is a group of organ-specific immune disorders characterized by an inflammatory process, which includes increased CD4^+^ T cells infiltration in the eyes (Caspi et al., 1986; Muhaya et al., 1999; Ilhan et al., 2008), and is usually activated by DCs. Abnormal DC activation is a major cause of the pathogenesis of uveitis (Xu et al., 2007; Heuss et al., 2012; Constantino-Jonapa et al., 2020). Thus, regulating DC activation may reverse the inflammatory state of this disease by influencing either DC-T contact or T cell activation. Exploring the regulatory factors on DC function and DC-T contact will help identify new therapeutic targets for this disease.

In the current study, we identify a regulatory pathway of sCD83 on DC-T contact formation. We demonstrate that sCD83 inhibits IS formation of DC-Ts by disrupting the distribution of F-actin and MHC-II at DC-T contacts, and we find that this inhibitory effect of sCD83 on DCs is via suppression of Rab1a, which controls the LRRK2 and F-actin colocalization. Furthermore, sCD83-treated DCs alleviates the symptoms of EAU and decreases the number of T cells in the eyes and lymph nodes of mice with EAU. Our findings provide a possible mechanism of sCD83 on DC-T contact by Rab1a-mediated F-actin and MHC-II localization and may provide a new therapeutic approach for the treatment of EAU.

## Materials and Methods

### Animals and EAU Model

Pathogen-free female C57BL/6 (6–8-week-old) mice were purchased from Beijing Vital River Laboratory Animal Technology Co., Ltd. (Beijing, China). These mice were maintained in specific pathogen-free conditions according to the guide for the care and use of laboratory animals of Shandong First Medical University & Shandong Academy of Medical Sciences (Jinan, China). The experiments were approved by the ethics committee of Shandong First Medical University & Shandong Academy of Medical Sciences (Jinan, China). The induction of EAU in C57BL/6 mice has been described in previous reports (Thurau et al., 1997; Beibei Wang et al., 2017; Lin et al., 2017). Briefly, C57BL/6 mice were subcutaneously immunized with 350 μg of human interphotoreceptor retinoid-binding protein peptide_1−20_ (IRBP_1−20_, China Peptides Co., Ltd., Suzhou, Jiangsu, China) that was emulsified in the complete Freund's adjuvant (Sigma–Aldrich Company, MA, USA). Concurrently, a single dose of 500 ng of pertussis toxin (PTX, Enzo Life Sciences, Farmingdale, NY, USA) was injected intraperitoneally. After immunization for 21 days, the mice were examined by histopathological examination or immunofluorescence examination, and the degree of disease was evaluated by a scoring system according to previously described (Thurau et al., 1997; Harimoto et al., 2014).

### sCD83 Construction and Usage

Mouse sCD83 protein consists of the extracellular domain of the membrane-bound CD83 (mCD83) molecule (22–133 aa), which was purified from the supernatant of transiently transfected eukaryotic cells (Roman-Sosa et al., 2016). Purified sCD83 was detected by sodium dodecyl sulfate–polyacrylamide gel electrophoresis (SDS-PAGE) and Western blot (Supplementary Figure 1). For animal treatment, sCD83 (10 μg/mouse) was used on day 8 after EAU immunization and was administered intravenously every other day. The eyes were harvested on 21 days for immunofluorescence staining or hematoxylin-eosin stain. The eyes and lymph nodes were harvested and prepared for flow cytometry. For cell stimulation, sCD83 (100 ng/mL) was added to the culture of isolated wild-type (WT) DC or DC2.4 cell line for pretreatment 24 h, and then the cells were collected for detection. Human immunoglobulin G (IgG) protein was used as a negative control.

### Plasmid Construction

Rab1a^Q70L^ (a GTP-restricted mutant, activated form) was generated by polymerase chain reaction site-directed mutagenesis and confirmed by sequencing. The pCAG-LifeAct-RFP plasmid was used for F-actin visualization in DCs according to the manufacturer's instructions (ibidi, Martinsried, Germany).

Non-targeting control shRNA and targeting shRNA were constructed by JiMan Sigma (Shanghai, China). The sequence of effective Rab1a shRNA is 5′-GCACAATTGGTGTGGATTT-3′, and the sequence of MD-2 shRNA is 5′-AGTTATTGTGATCACTTGA-3′, respectively. They were constructed into PGMLV-SC5, separately. The cells were then transfected with 5–20 nM PGMLV-SC5-Rab1a shRNA or non-targeting control shRNA (NC) as per the protocol provided by the manufacturer. The pCAG-LifeAct-RFP plasmid was used for F-actin visualization in DCs according to the manufacturer's instructions (ibidi, Martinsried, Germany). pCAG-RFP empty plasmid was used as control.

### Cells Culture and Isolation

DC2.4 cell line (a kind gift from Professor Yiwei Chu, Fudan University, Shanghai, China) was cultured in RPMI-1640 containing 10% fetal bovine serum and double antibodies. These cells were stimulated with IRBP_1−20_ (10 ng/mL) and PTX (10 ng/mL) overnight.

WT DCs or CD4^+^ T cells were obtained from the spleen and selected using a CD4-negative and CD11c-positive selection kit (Miltenyi Biotec, Bergisch Gladbach, Germany), respectively. Splenocytes were obtained, and red blood cell lysis buffer (Solarbio Science & Technology Co., Ltd., Beijing, China) was used to obtain the single-cell suspension as previously reported (Shao et al., 2003; Lin et al., 2017). And then, CD4^+^ T cells and DCs were isolated by kits according to manuscript protocol.

### DC-T Contact Detected by Immunofluorescence

Wild-type DCs or DC2.4 cell lines were incubated with IRBP_1−20_ (10 ng/mL) and PTX (5 ng/mL) overnight to be activated (Lin et al., 2018). sCD83 (10 ng/mL) were added to stimulate overnight. CD4^+^ T cells were purified from the spleen of IRBP_1−20_-immunized B6 mice and were stimulated with IRBP_1−20_ (10μg/mL) in the presence of 1 × 10^7^ irradiated syngeneic spleen cells as antigen presenting cells (APCs) in the presence of IL-2 (10 ng/mL), and then antigen-specific CD4^+^ T cells were obtained by CD4^+^ magnetic beads (Miltenyi Biotec, Bergisch Gladbach, Germany). CD4^+^ T cells were cocultured with antigen-pulsed mature DCs (T cell:DC ratio = 10:1) overnight. And then, the cells were fixed in phosphate-buffered saline (PBS)/4% paraformaldehyde for 10 min, followed by incubation with PBS/0.1 M of glycine for 3 min. Cells were permeabilized with 0.1% Triton X-100 for 20 min and then blocked with 2% bovine serum albumin buffer for 20 min. Next, the cells were stained with a 1:500 dilution of TRITC phalloidin (Molecular Probes, Carlsbad, CA, USA) as previously reported (Quintana et al., 2009; Lin et al., 2015), or cells were stained with anti-Rab1a (Abcam, Cambridge, MA, USA), anti-MHC-II (R&D Systems, Inc., Minneapolis, MN, Canada), and anti-LRRK2 (Abcam, Cambridge, MA, USA) antibody (1:1,000 dilution) for 60 min. After washing three times, corresponding fluorescent antibodies were added and detected by a confocal microscope.

### Confocal Imaging

Images of the cells were taken with a confocal microscope (LMS 780, Zeiss, Germany) equipped with an APO oil immersion objective lens (63 ×, NA = 1.40) (Lin et al., 2015). The images were analyzed with the Imaris Software (Bitplane AG, Zurich, Switzerland) and ImageJ (National Institutes of Health, Bethesda, MD, USA). DC-T contact was observed by *z*-axis scanning. Three-dimensional (3D) intercellular contacts were reconstructed and analyzed by Imaris Software and ImageJ. Total fluorescence value of MHC class II or F-actin was quantified after 3D image reconstructed. Colocalization of Rab1a and LRRK2, or Rab1a and MHC-II, in DCs were analyzed by ImageJ with colocalization index (Pearson correlation coefficient, Pearson's *r*).

### Rab1a Activation Assay

Cells were lysed with ice-cold lysis buffer [50 mM Tris-HCl (pH 7.5), 2% Nonidet P-40, 10 mM MgCl_2_, 300 mM NaCl, and protease inhibitor]. Collected lysates were incubated with GTP-agarose beads (Novus Biologicals, Co, USA) at 4°C for 2 h. And then, the beads were collected by centrifugation, and the binding of Rab1a was analyzed by Western blot with anti–Rab1a mAb.

### Flow Cytometry

Aliquots of 1 × 10^6^ cells were stained with different monoclonal antibodies according to standard protocols. The cells were analyzed on a FACSVerse cytometer (BD Biosciences, San Diego, CA, USA). Fluorescent antibodies of CD45 (clone 30-F-11), CD3ε (clone 145-2C11), CD4 (clone GK1.5), CD83 (clone Michel-19), CD11b (clone M1/70), ly6c(clone HK1.4), F4/80 (clone BM8), B220 (clone RA3-6B2), NK1.1 (clone PK136), MHC-II (clone M5/114.15.2), CD11c (clone N418), CD69 (clone H1.2F3), and Ki67 (clone B56) conjugated with the corresponding fluorescent dyes (eBioscience, San Diego, CA, USA) were used in the experiments.

### Western Blot

DC2.4 cells or sCD83-treated DC2.4 cells were lysed with RIPA buffer (Beyotime Biotechnology, Shanghai, China). Identical quantities of protein were separated by 10% SDS-PAGE and transferred to polyvinylidene difluoride membranes. Subsequently, 5% non-fat dry milk in Tris-buffered saline 0.1% Tween 20 (TBS-T) was used to block non-specific binding sites for 1 h. After washing with TBS-T, membranes were incubated with primary antibodies against mouse Rab1a (Abcam, Cambridge, MA, USA), LRRK2 (Abcam, Cambridge, MA, USA), F-actin, MHC-II (R&D Systems, Inc., Minneapolis, MN, USA), and GAPDH (Cell Signaling Technology, Beverly, MA, USA) at 4°C for overnight. Membranes were then washed and incubated with secondary antibodies (goat-anti-rabbit IgG antibodies) conjugated to horseradish peroxidase (Beyotime Biotechnology, Shanghai, China) for 1 h. Finally, the membranes were developed using the Super Signal West Pico Chemiluminescent Substrate (Thermo Scientific, Rockford, IL, USA). Densitometric analyses were performed using the ImageJ software (National Institutes of Health, Bethesda, MD, USA).

### Statistical Analysis

Data analysis was performed using GraphPad Prism 5 (GraphPad Software, San Diego, CA, USA). Two-tailed Student *t*-test or one-way analysis of variance was used as parametric tests. Mann–Whitney *U*-test or Kruskal-Wallis test were used as nonparametric tests. Data were represented as mean ± SEM. *p* < 0.05 (^*^), 0.01 (^**^), and 0.001 (^***^) were considered to be significant.

## Results

### sCD83 Decreases DC-T Synapse Formation by Reducing Assembly of F-Actin at Sites of DC-T Contact

**Figure 1:**
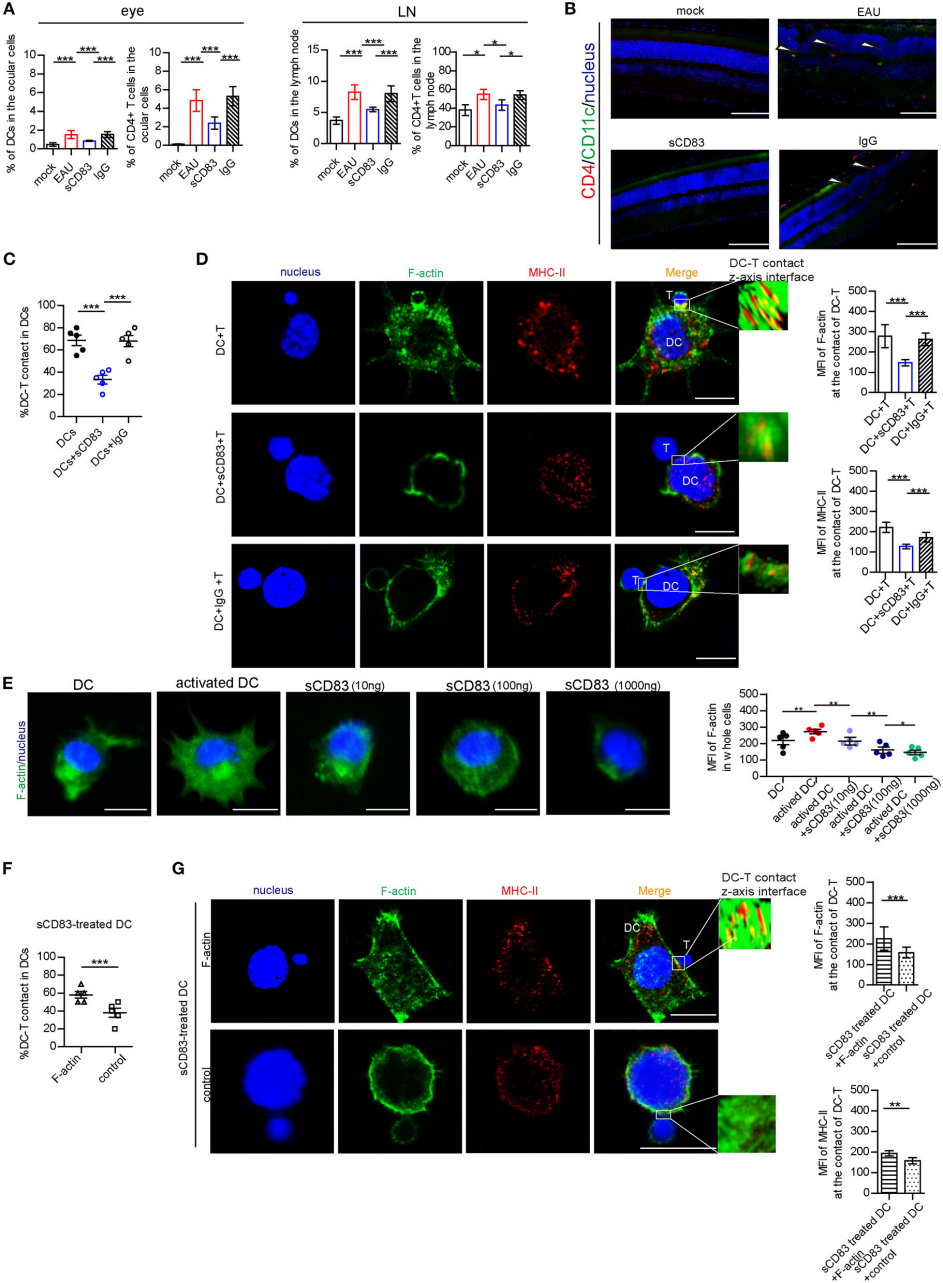
The effect of sCD83 on T cells and DCs *in vivo* and in *in vitro* experiment. **(A)** sCD83 treatment decreased the increased percentage of CD11c^+^ MHC-II^+^ DCs and CD4^+^ T cells in eyes and lymph nodes of EAU mice. These data are from three separate experiments; five mice were used for every group and shown as mean ± SEM. *p < 0.05, ***p < 0.001. **(B)** The retinal lesions (white arrow showed multiple protrusions were found in the outer nuclear layer), CD4^+^ T cells (red). and CD11c^+^DC lymphocyte subpopulation (green) in the eyes of mock, EAU, and sCD83-treated EAU mice were detected by immunofluorescence. Nucleus is blue. Bar = 100 μm. **(C)** sCD83 treatment decreases the percentage of DC2.4-T contacts. **(D)** sCD83 treatment also decreases the IS formation of DC2.4-T contacts that require both F-actin and MHC-II (left panel). Mean fluorescence value of F-actin and MHC-II at DC-T contacts (right panel). Five cell–cell contacts/group were analyzed by confocal and Imaris Software. DC-T contact was reconstructed and analyzed by Imaris Software. The middle section or the largest section of the *z*-axis was selected and displayed in the right panel. Total fluorescence value of MHC class II or F-actin was quantified after 3D image reconstructed. **(E)** The distribution (left panel) and the mean value of F-actin (right panel) in DC2.4, which was under different concentrations of sCD83 stimulation. Five cell–cell contacts/group were analyzed. **(F)** Overexpression of F-actin into sCD83-treated DCs increased the percentage of DC2.4-T contact in DCs. **(G)** The distribution (left panel) and the mean value of F-actin and MHC-II (right panel) in F-actin overexpressed sCD83-treated DCs compared to empty plasmid overexpressed sCD83-treated DCs (control). Five cell–cell contacts/group were analyzed. Data of **(C–F)** are shown as mean ± SEM. *p < 0.05, **p < 0.01, ***p < 0.001. Bar = 5μm.

As previously reported (Lin et al., 2018), sCD83 treatment decreases the symptoms in EAU. In our study, we found that sCD83 treatment decreased the increased percentage of CD11c^+^ MHC-II^+^ DCs and CD4^+^ T cells in the eyes and lymph nodes of infected mice (Figure 1A). The retinal lesions of each group and T and DC lymphocyte subpopulation imaging are shown in Figure 1B. In the eyes of EAU, multifocal retinal fold (white arrows) and CD4^+^ T cells (red) and DCs (green) infiltration were found (Figure 1B), and DC-T contacts were found in lymph nodes of EAU (Supplementary Figure 2). However, almost no retina damage and the less lymphocytes infiltration were found in the eyes of sCD83-treated EAU mice (Figures 1A,B). Based on *in vitro* experiments, sCD83-treated DCs formed fewer DC-T contacts than those without treatment (Figure 1C). With sCD83 treatment, the mean fluorescence value of F-actin and MHC-II at DC-T contacts decreased (Figure 1D), and F-actin lost to accumulate and around with MHC-II to form DC-T synapse (Figure 1D). F-actin and MHC-II were essentially diffused at the sCD83-treated DC-T contact (Figure 1D). As a result, sCD83 treatment disrupts IS formation that requires both F-actin and MHC-II. However, sCD83 treatment did not influence the activation of T cells and did not influence sCD83-treated T cell contact with DCs (Supplementary Figure 3).

Further, the mean fluorescence value of F-actin increased in activated DCs (Figure 1E). However, under different concentrations of sCD83 stimulation, we found that F-actin fluorescence in DCs gradually reduced with increased sCD83 concentration (Figure 1E). In addition, disrupting F-actin with cytochalasin D reduced DC-T contact and MHC-II accumulation at DC-T contacts (Supplementary Figure 4), whereas overexpression of F-actin in DCs promoted DC-T contact and MHC-II accumulation at DC-T contacts (Supplementary Figure 4). Overexpression of F-actin rescued the negative effect of sCD83 on DCs, causing increased DC-T contact, and F-actin and MHC-II accumulation at DC-T contacts (Figures 1F,G). Thus, sCD83 regulates DC-T synapse formation and MHC-II accumulation at DC-T contacts by controlling F-actin in DCs.

### sCD83 Decreases Rab1a Expression in DCs

**Figure 2:**
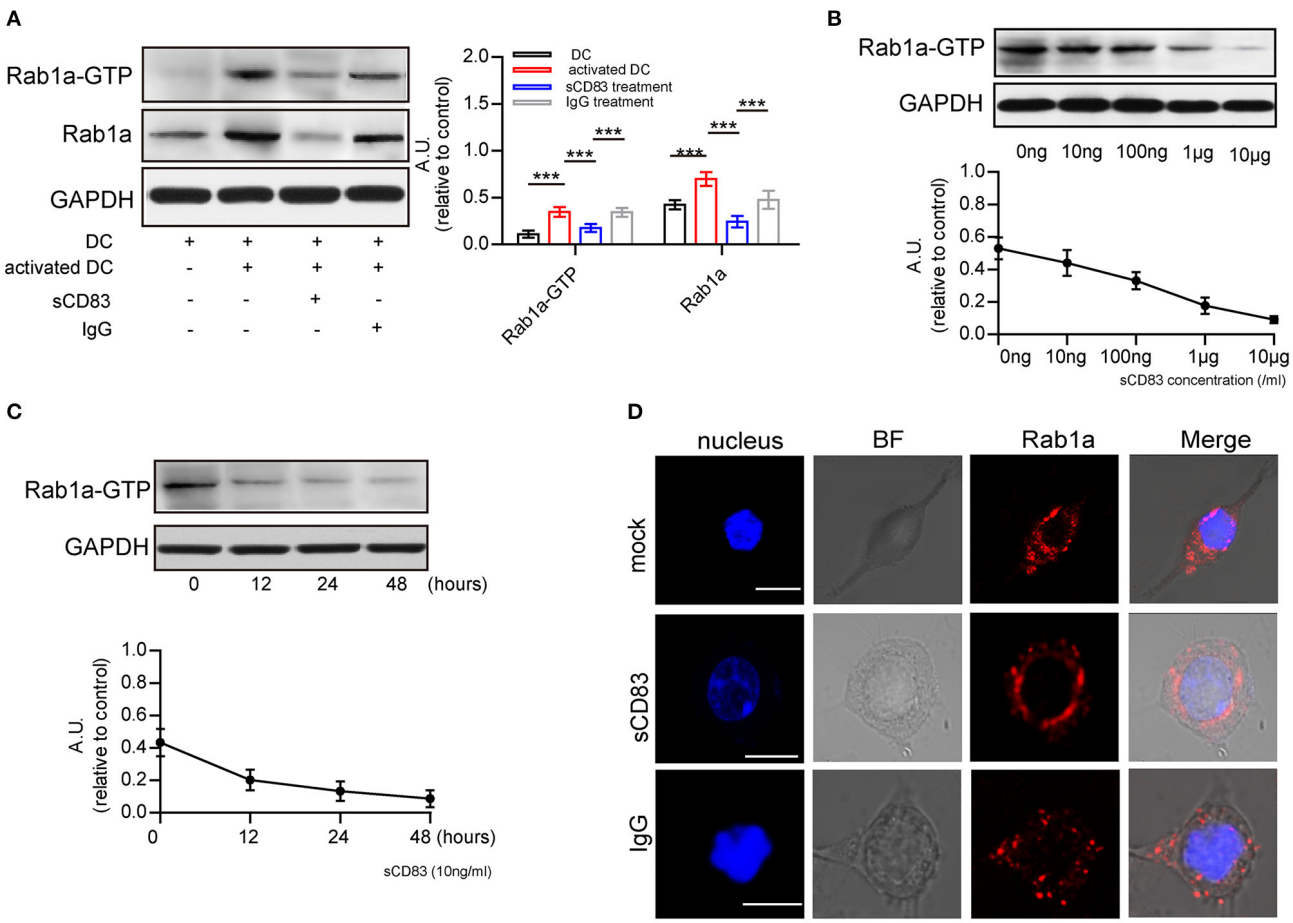
sCD83 inhibits the expression of Rab1a-GTP and the distribution of Rab1a. **(A)** The expression of Rab1a and Rab1a-GTP in DC2.4, activated DC2.4 and sCD83-treated activated DC2.4. **(B)** With different concentrations of sCD83 treatment for 24 h, the expression of Rab1a-GTP in activated DC2.4 was analyzed by Western blot. **(C)** With 10 ng/mL sCD83 treatment for 6, 12, or 24 h, the expression of Rab1a-GTP in activated DCs was analyzed by Western blot. Data of **(A–C)** are from three separate experiments and shown as mean ± SEM. ***p < 0.001. **(D)** The distribution of Rab1a (red) in activated DC2.4 cells, sCD83-treated activated DC2.4 cells, and IgG-treated activated DC2.4 cells. Nucleus is blue. Bar = 5μm.

Next, we investigated the possible mechanism underlying sCD83-mediated regulation of F-actin at DC-T contacts. F-actin assembly is controlled by activated Rab1a (Kicka et al., 2011). As a small G protein, Rab1a is activated by binding with GTP. Using DCs incubated with Rab-GTP-agarose beads and an anti-Rab1a antibody to detect Rab1a binding, we found following PTX and IRBP stimulation a significant increase in the GTP-binding activity of Rab1a, as well as Rab1a in DCs (Figure 2A), Both of Rab1a and Rab1a-GTP decreased in response to sCD83 stimulation (Figure 2A). Moreover, we found that GTP-binding activity of Rab1a decreased following sCD83 administration at high concentrations (Figure 2B), as well as in response to the time of sCD83 action (Figure 2C). Thus, these findings indicate that sCD83 affects the amount of GTP-binding activity of Rab1a. Moreover, following sCD83 treatment, we found that the average fluorescence of Rab1a decreased and that localization was mainly around the nucleus (Figure 2D). Based on these findings, we conclude that sCD83 influences the expression and cellular localization of Rab1a in DCs.

### sCD83 Disrupts LRRK2/F-Actin Distribution in DCs Through Rab1a

**Figure 3:**
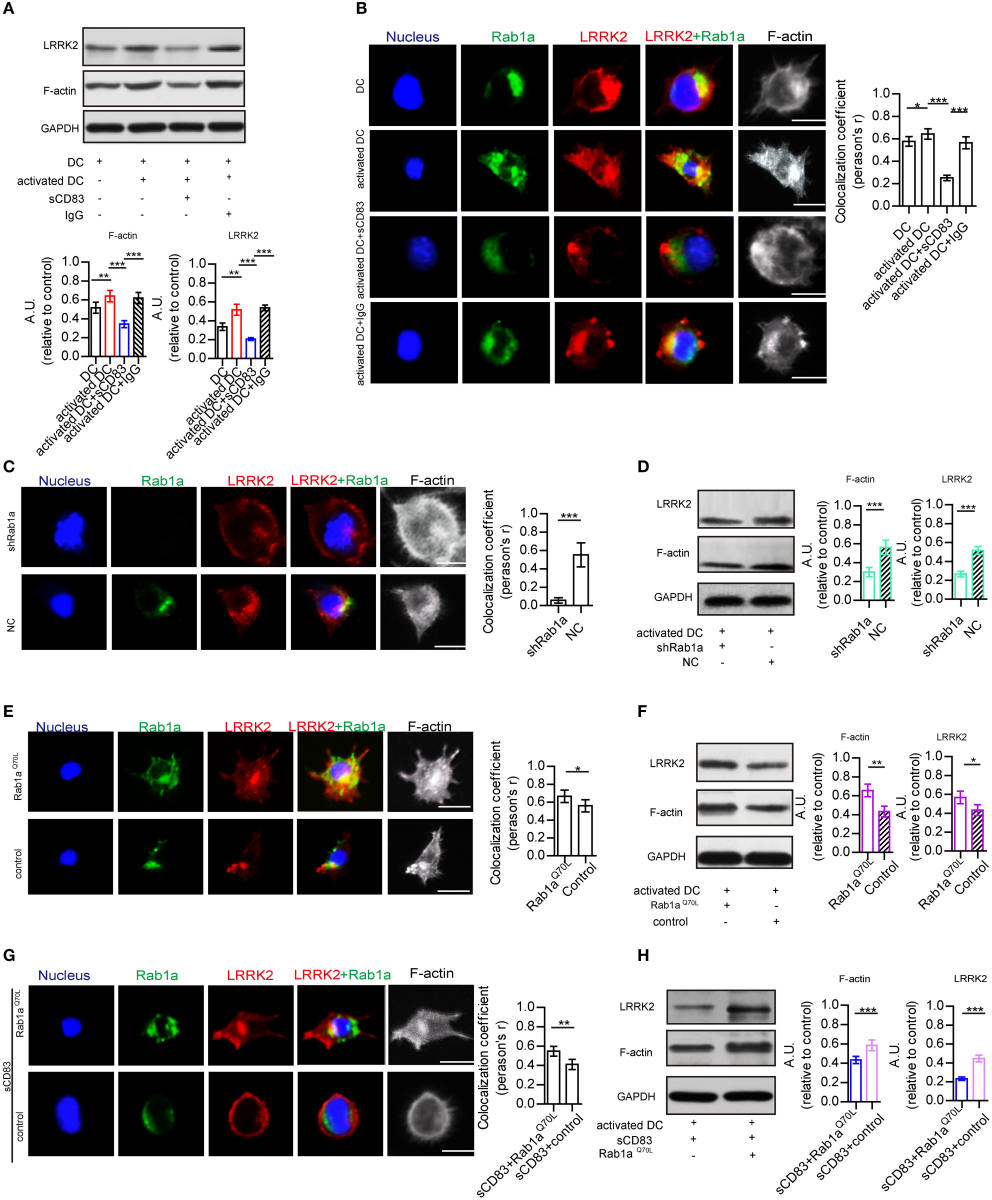
Rab1a plays an important role in regulating the effect of sCD83 on the F-actin in DCs. **(A)** The expression of F-actin and LRRK2 in the DC2.4, activated DC2.4 cells, activated DCs with sCD83 treatment, or activated DCs with IgG treatment. **(B)** The localization of Rab1a (green), LRRK2 (red), and F-actin (white) in DC2.4 cells or activated DC2.4 cells, sCD83 treated-activated DCs, and IgG-treated activated DCs. Colocalization index (Pearson'*r*) was graphically represented (right panel). **(C)** With shRab1a or NC treatment, the localization of Rab1a (green), LRRK2 (red), and F-actin (white) in DC2.4 cells. Colocalization index (Pearson'*r*) was graphically represented (right panel). **(D)** The expression of F-actin and LRRK2 in activated DC2.4 cells with shRNA Rab1a (shRab1a) treatment, or activated DCs with NC treatment. **(E)** The localization of Rab1a (green), LRRK2 (red), and F-actin (white) in Rab1a^Q70L^-treated DC2.4 cells, or empty plasmid-treated DC2.4 cells (control). Colocalization index (Pearson'*r*) was graphically represented (right panel). **(F)** The expression of F-actin and LRRK2 in activated DC2.4 cells with Rab1a^Q70L^ treatment or empty plasmid treatment (control). **(G)** Overexpressed Rab1a^Q70L^ (green) or empty plasmid (control) influence the distribution of F-actin (white) and LRRK2 (red) in DC2.4 cells. Colocalization index (Pearson'*r*) was graphically represented (right panel). **(H)** Overexpressed Rab1a^Q70L^ promotes the expression of F-actin and LRRK2 in sCD83-treated DCs, but empty plasmid (control) could not. Nucleus is blue. Bar = 5μm. Data of **(A,D,F,H)** were from three separate experiments, and data of **(B,C,E,G)** were form six cells/group and shown as mean ± SEM. *p < 0.05, **p < 0.01, ***p < 0.001.

As Rab1a regulates localization of members of the LRRK2-related kinase family (Kicka et al., 2011), which controls the actin cytoskeleton of DCs, we hypothesized that sCD83 regulates DC morphology by affecting the expression and localization of F-actin and LRRK2 in DCs through Rab1a. First, we evaluated the expression and localization of Rab1a, F-actin, and LRRK2 in DCs with and without sCD83 treatment. Using PTX and IRBP stimulation, we found increased expression of F-actin and LRRK2 in activated DCs (Figure 3A). We also found that activated DCs were morphologically elongated in multiple directions, with F-actin distributed and supporting multiple directions, and Rab1a and LRRK2 were colocalized in these DCs (Figure 3B). Following sCD83 treatment, the expression of F-actin and LRRK2 decreased (Figure 3A), and Rab1a and LRRK2 were no longer colocalized so well (Figure 3B), and the distribution of F-actin was disrupted.

To further investigate the role of Rab1a in regulating the distribution of LRRK2 and F-actin in DCs, shRNA-Rab1a was used to interfere with Rab1a expression (Supplementary Figure 5). Following shRNA-Rab1a treatment, we found that DCs became morphologically round with short spikes (Figure 3C). The expression of F-actin and LRRK2 decreased in shRNA Rab1a-treated DCs (Figure 3D). In contrast, overexpression of an activated form of Rab1a (Rab1a^Q70L^) resulted in morphological changes of DCs, which became elongated with multiple extensions (Figure 3E). Rab1a colocalized with LRRK2 in Rab1a^Q70L^-treated DCs (Figure 3E), and LRRK2 and F-actin were uniformly distributed in cells and supported extended spike formation (Figure 3E). Further, Rab1a^Q70L^ overexpression increased expression of F-actin and LRRK2 (Figure 3F). These findings indicate that activation of Rab1a correlates with the expression and localization of LRRK2 and F-actin in DCs.

Next, to determine whether the effect of sCD83 on DCs was regulated by Rab1a, Rab1a^Q70L^ was overexpressed in sCD83-treated DCs for 72 h. We found that Rab1a^Q70L^ overexpression promoted F-actin and LRRK2 expression and colocalization of Rab1a and LRRK2 in sCD83-treated DCs (Figures 3G,H). Based on these findings, we concluded that Rab1a participated in the regulation of sCD83 on F-actin in DCs by controlling colocalization of Rab1a and LRRK2.

### Rab1a Controls Expression and Localization of MHC-II on the Surface of DCs

**Figure 4:**
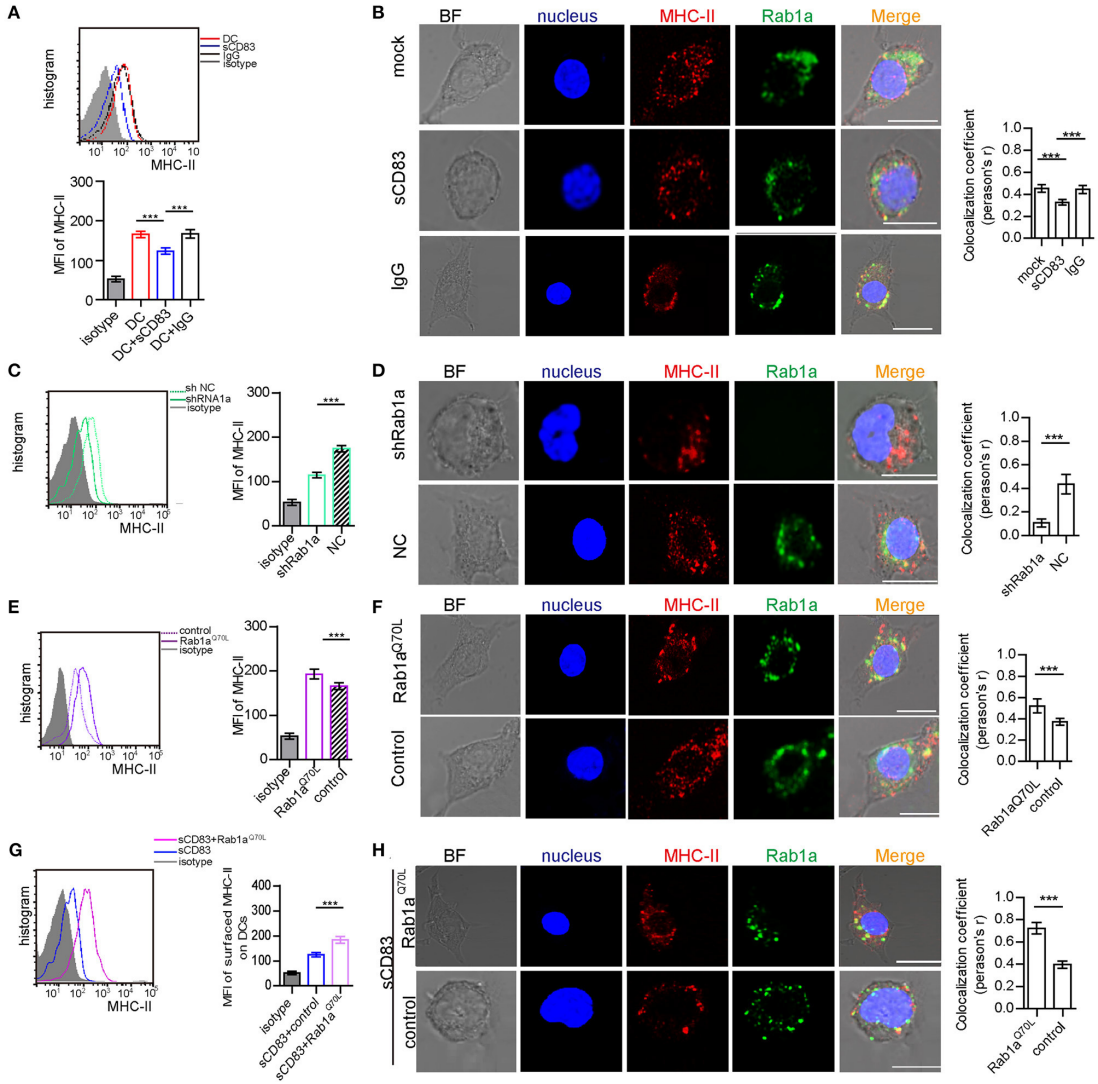
Rab1a controls expression and localization of MHC-II on the surface of DCs. **(A)** The expression of MHC-II on the surface of activated DC2.4, sCD83-treated activated DC2.4, or IgG-treated activated DC2.4 cells. **(B)** The localization of MHC-II (red) and Rab1a (green) in activated DC2.4, sCD83-treated activated DC2.4, and IgG-treated activated DC2.4. Colocalization index (Pearson'*r*) was graphically represented (right panel). **(C)** The expression of MHC-II on the surface of shRNA Rab1a-treated activated DC2.4, compared to NC-treated activated DC2.4. **(D)** The localization of MHC-II (red) and Rab1a (green) in shRNA Rab1a-treated or NC-treated activated DC2.4. Colocalization index (Pearson'*r*) was graphically represented (right panel). **(E)** The expression of MHC-II on the surface of Rab1a^Q70L^ overexpressed activated DC2.4, compared to empty plasmid transfected activated DC2.4 (control). **(F)** The localization of MHC-II (red) and Rab1a (green) in Rab1a^Q70L^ overexpressed activated DC2.4 or empty plasmid transfected activated DC2.4. Colocalization index (Pearson'*r*) was graphically represented (right panel). **(G)** The expression of MHC-II on the surface of Rab1a^Q70L^ overexpressed sCD83-treated activated DC2.4, compared to empty plasmid transfected sCD83-treated activated DC2.4 (control). **(H)** The localization of MHC-II (red) and Rab1a (green) in Rab1a^Q70L^ overexpressed sCD83-treated DC2.4 or empty plasmid transfected sCD83-treated activated DC2.4 (control). Colocalization index (Pearson'*r*) was graphically represented (right panel). Bar = 5μm. Data are from three separate experiments; six cells/group were used and shown as mean ± SEM. ***p < 0.001.

As a central molecule of IS, MHC-II expression in sCD83-treated DC-T contacts decreased and no longer accumulated in synapses (Figure 1D). In addition, sCD83 treatment decreased the surface expression of MHC-II on DCs (Figure 4A). And MHC-II in sCD83-treated DCs prefers to accumulate around the nucleus (Figure 4B). In activated DCs, MHC-II can colocalize with Rab1a (Figure 4B). However, sCD83 treatment disrupted the colocalization of MHC-II and Rab1a (Figure 4B). Based on these findings, sCD83 influences the expression and localization of MHC-II in DCs. We next investigated to determine whether the regulatory effect of sCD83 on MHC-II expression and localization in DCs was controlled by Rab1a, which is involved in vesicle transport, a process that transports proteins to the cell membrane. Following shRNA-Rab1a treatment, we found that MHC-II expression decreased (Figure 4C), and MHC-II accumulated around the nucleus of DCs (Figure 4D). In contrast, overexpression of Rab1a^Q70L^ increased MHC-II expression (Figure 4E), and MHC-II preferred to colocalize with Rab1a in these DCs (Figure 4F). Moreover, overexpression of Rab1a^Q70L^ in sCD83-treated DCs also increased MHC-II expression on the surface of DCs and promoted MHC-II colocalized with Rab1a (Figures 4G,H). Thus, these findings indicate that sCD83 influences the localization of MHC-II in DCs by Rab1a.

### Inhibitory Effect of sCD83 on DC-T IS Formation Is Regulated by Rab1a-Mediated F-Actin

**Figure 5:**
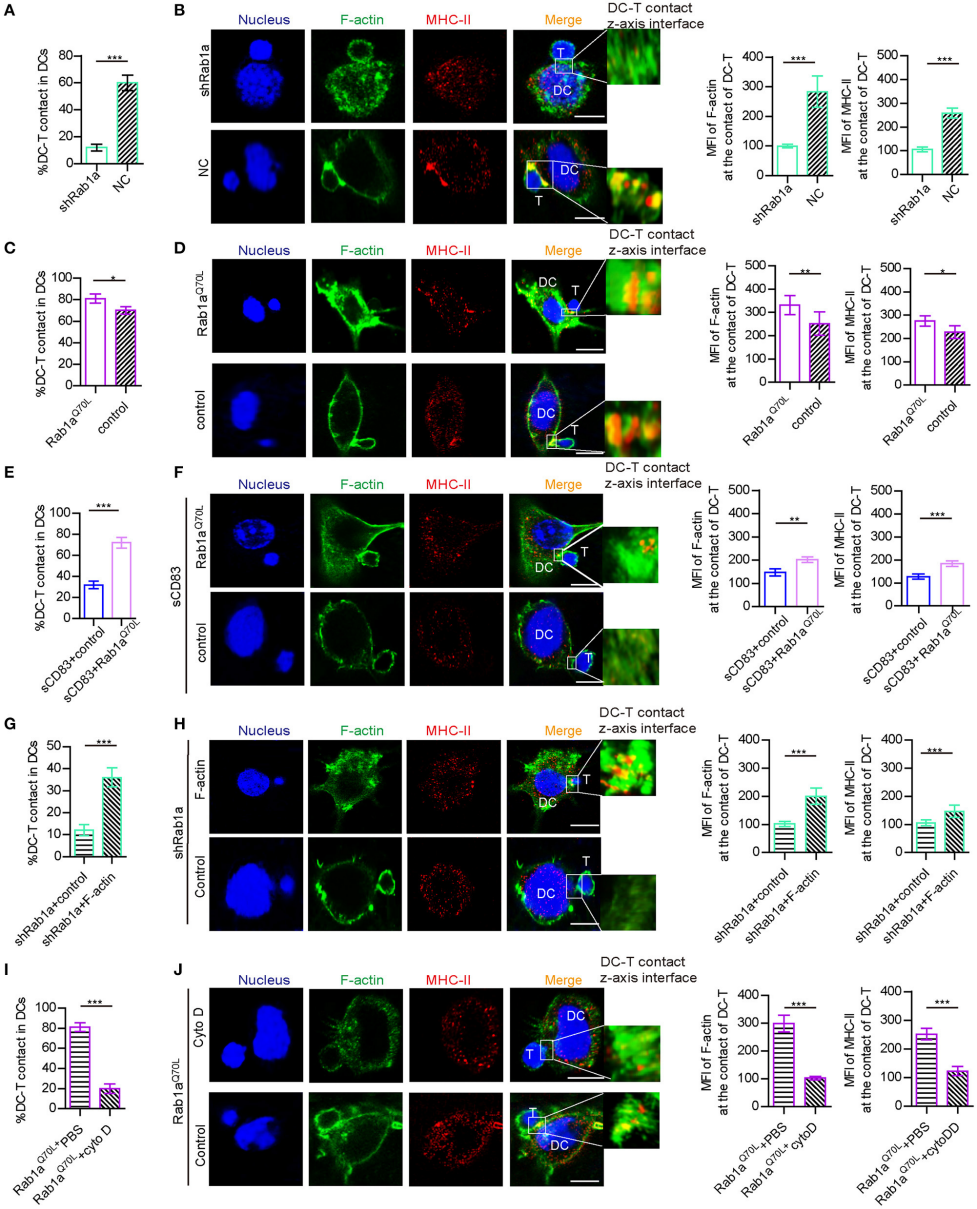
Rab1a-mediated F-actin regulated IS formation between DC and T cells. **(A)** With shRNA Rab1a or NC treatment, the percentage of DC-T contact in DCs. **(B)** With shRNA Rab1a, or NC treatment, the distribution (left panel) and mean fluorescence value (right panel) of F-actin and MHC-II at the contact between DC 2.4 and T cells. **(C)** Overexpression of Rab1a^Q70L^ in DC promotes the percentage of DC-T contacts and **(D)** promotes the accumulation (left panel) and mean fluorescence value (right panel) of F-actin and MHC-II at the contacts of DC-T, compared to the empty plasmid transfected DC. **(E)** Overexpression of Rab1a^Q70L^ in sCD83-treated DC promotes the percentage of DC-T contacts and **(F)** promotes the accumulation (left panel) and mean fluorescence value (right panel) of F-actin and MHC-II at the contacts of DC-T, compared to the empty plasmid transfected sCD83-treated DC. **(G)** Overexpression of F-actin in shRab1a-treated DC promotes the percentage of DC-T contacts and **(H)** promotes the accumulation (left panel) and mean fluorescence value (right panel) of F-actin and MHC-II at the contacts of DC-T, compared to the empty plasmid transfected shRab1a-treated DC (control). **(I)** Cytochalasin D treatment decreased the percentage of DC-T contact (left panel) in Rab1a^Q70L^ overexpressed DCs, compared to PBS-treated Rab1a^Q70L^ overexpressed DCs. **(J)** Cytochalasin D treatment distributed the accumulation (left panel) and mean fluorescence value (right panel) of F-actin and MHC-II in Rab1a^Q70L^ overexpressed DCs, compared to PBS (control)–treated Rab1a^Q70L^ overexpressed DCs. Bar = 5μm. Data of **(A,C,E,G,I)** are from three separate experiments, and data of **(B,D,F,H,J)** were from five cells/group and were shown as mean ± SEM. *p < 0.05, **p < 0.01, ***p < 0.001.

Because Rab1a affects the expression and localization of F-actin and MHC-II, which are essential for DC-T IS formation, we explored whether Rab1a may be a regulator of DC-T synapse formation. Following shRNA-Rab1a treatment, we found that DC-T contact decreased, and IS formation between DC-T was disrupted (Figures 5A,B). However, overexpression of Rab1a^Q70L^ increased the percentage of DC-T contact and promoted IS formation to accumulate F-actin and MHC-II at DC-T contact (Figures 5C,D). Based on these findings, we conclude that Rab1a regulates DC-T synapse formation.

In addition, overexpression of Rab1a^Q70L^ rescued the expression and altered localization of MHC-II in sCD83-treated DCs (Figures 3G,H). Overexpression of Rab1a^Q70L^ also increased the percentage of DC-T contacts and promoted MHC-II relocation to contacts of sCD83-treated DC-T (Figures 5E,F). Based on these findings, we conclude that sCD83 influences localization of MHC-II by Rab1a and that the inhibitory effect of sCD83 on IS formation between DC-Ts is regulated by Rab1a.

Furthermore, we found that overexpression of F-actin in shRNA Rab1a-treated DCs partially increased the percentage of DC-T contact and promoted IS formation between DC-T (Figures 5G,H). Following overexpression of Rab1a^Q70L^ in DCs for 72 h and then disruption of F-actin arrangement by cytochalasin D, DC-T contact formation decreased, and cells lost the ability to form IS (Figures 5I,J). Therefore, Rab1a regulates DC-T contact and DC-T IS by F-actin.

### sCD83-Treated DCs Decrease T Cell Number in EAU Mice

**Figure 6:**
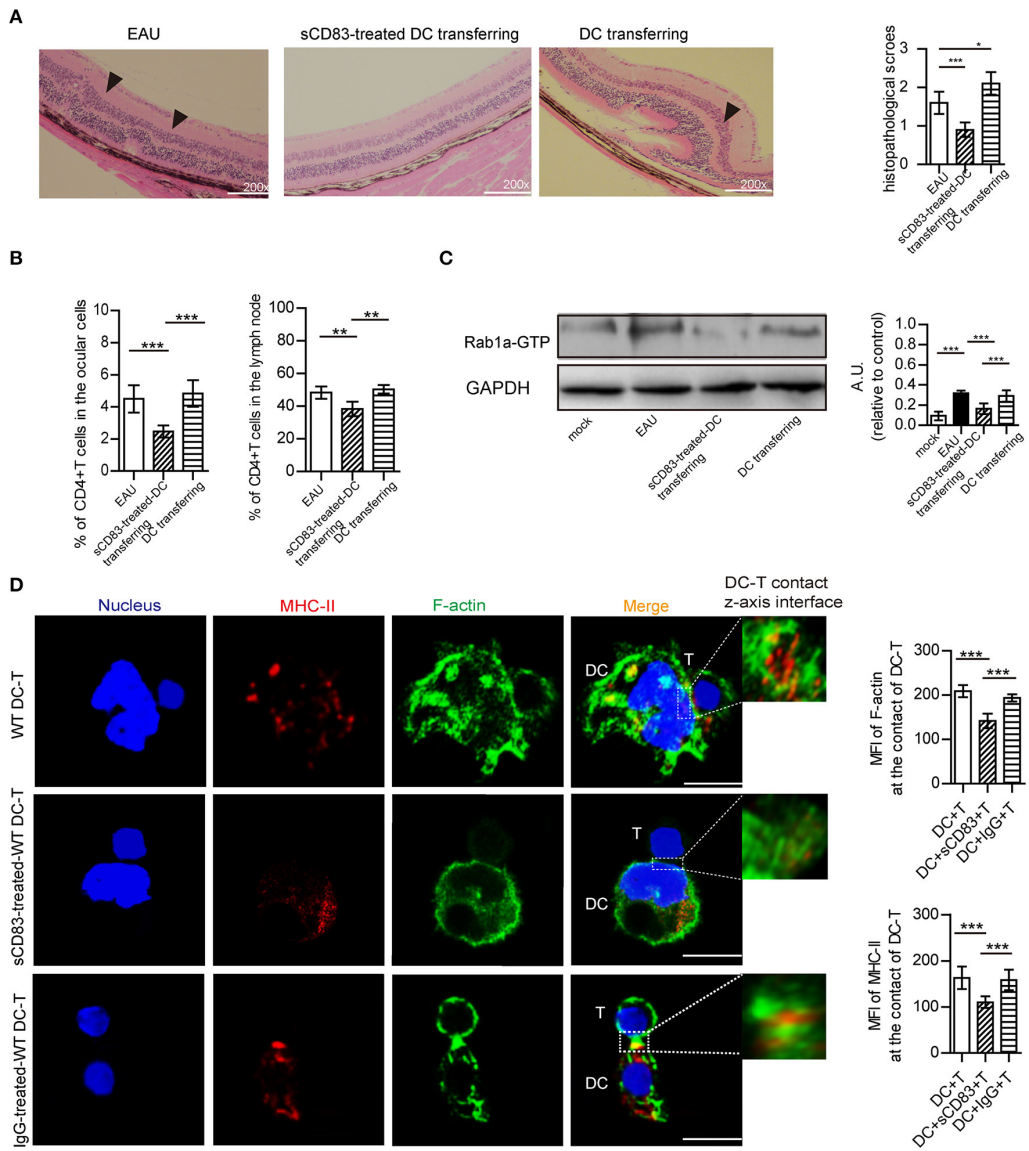
The effect of sCD83-treated DCs on EAU and the synapse formation between sCD83-treated DCs and T cells *in vitro* experiment. **(A)** Representative images of the retina from an EAU, EAU with sCD83-treated DCs transferring, and EAU with activated DCs transferring as assessed by histology. Hematoxylin-eosin staining of the retina at 200× magnification. Black arrows mark retinal disorganization. Scale bar = 100μm. The histopathological scores were evaluated in three mice every group (right panel). **(B)** The percentage of T cells in the eyes and lymph nodes of these mice with sCD83-treated DCs transferring or activated DCs transferring or not. **(C)** The expression of Rab1a-GTP in DCs from mock, EAU, and EAU with sCD83-treated DCs transferring. **(D)** sCD83 treatment reduces the expression and localization of MHC-II and F-actin at the site of DC-T contacts *in vitro* experiments. Bar = 5μm. Data of **(A–C)** were from three separate experiments, and three mice/group were used. Data of **(D)** are from three separate experiments and shown as mean ± SEM. *p < 0.05, **p < 0.01, ***p < 0.001.

We then prepared and transferred sCD83-treated DCs to a mouse model of EAU and found that the retinal damage of EAU was alleviated (Figure 6A), and there was a decreased number of T cells in the eyes and lymph nodes of these mice with sCD83-treated DCs transferring (Figure 6B). However, transferring activated DCs into EAU aggravated the retinal damage of EAU and increased the percentage of T cells in the eyes and lymph nodes of these mice (Figures 6A,B). Moreover, the expression of Rab1a-GTP in DCs from sCD83-treated DCs transferred to animals with EAU was lower than that found in controls and activated DCs transferred mice (Figure 6C). In addition, we found that sCD83 decreased the percentage of WT DC-T contacts as well as reduced the expression and localization of MHC-II and F-actin at the site of DC-T contacts *in vitro* experiments (Figure 6D). Thus, these findings demonstrate that sCD83-treated DCs alleviate symptoms in a mouse model of EAU by inhibiting DC-T contact.

## Discussion

The findings from our study show that sCD83 disrupts the accumulation of F-actin in DCs, resulting in decreased IS formation between DC-T, and that effect of sCD83 on DCs is mediated by decreased the expression of Rab1a to suppress the LRRK2/F-actin pathway and disrupt MHC-II localization on sites of DC-T contact (Supplementary Figure 6). In addition, our findings identify a new regulatory mechanism of DC-T contact formation. The activation and clonal expansion of naive T cells by antigen-loaded DCs are key events during immune response, and this activation involves the formation of a specialized IS between a mature DC and a CD4^+^ T cell (Dustin, 2009). Most prior studies on IS have focused on the T cell aspect of the synapse, with little information on the DC side (Rodriguez-Fernandez and Corbi, 2005; Rodriguez-Fernandez et al., 2009). The activation status of an encountered T cell controls the length of DC–T cell contact and drives cytoskeletal polarization in DC. In turn, disruption of DC IS formation blocks T cell activation (Al-Alwan et al., 2001). F-actin is an important cytoskeletal protein that clusters at DC-T contacts to maintain the structure and probably the signaling from this region (Al-Alwan et al., 2001). It also regulates DC synapse formation and function by promoting cell polarization and intracellular redistribution of proteins and organelles (Lin et al., 2015; Schulz et al., 2015). Disruption of the F-actin cytoskeleton in DCs severely diminishes T cell activation (Al-Alwan et al., 2001). In the current study, we found that sCD83 regulates F-actin by decreasing Rab1a activation. As a small G protein, activation of Rab1a may result in binding to Roco2 and further regulation of the activity of members of the Roco2 kinase family, including LRRK2, which controls F-actin rearrangement. In contrast to previous reports that found inhibitor cytokines such as IL-10–regulated F-actin in DCs (Xu et al., 2017), we showed an sCD83-based mechanism on regulating F-actin in DCs, a finding that contributes to a better understanding of the underlying regulatory mechanism of the DC cytoskeleton and DC-T contact.

Additionally, sCD83 regulated DC-T contact by decreasing Rab1a expression, which mediated endoplasmic reticulum (ER)–Golgi transport, and could result in the decrease of cell surface protein expression to influence DC-T contact. sCD83 may decrease the surface protein expression in DCs by Rab1a to inhibit DC-T contact and T cell activation. In this article, DCs were activated by PTX stimulation, which may increase cAMP (Burch et al., 1988) and up-regulate class II transcription in DCs. sCD83 may inhibit these activated DCs by down-regulating the expression of Rab1a to disrupt cAMP-mediated F-actin polymerization (Kicka et al., 2011).

As the extracellular domain of immunoglobulin CD83, sCD83 is absorbed by DCs (Yang et al., 2015) and may be degraded by Rab1a-mediated autophagy. sCD83 is secreted by activated CD83^+^ DC or other CD83^+^ cells, which may result in a large amount of sCD83 consumption or inhibition of Rab1a activity, leading to inhibition of the cytoskeleton and disruption of surface molecules in DCs, thereby preventing cell activation and function. This process would inherently act as a self-regulatory mechanism of sCD83 on DCs. Furthermore, sCD83 binds with MD-2 to inhibit the MD-2/TLR4 signaling pathway (Horvatinovich et al., 2017). However, MD-2 knockdown in DCs had no effect on the F-actin arrangement in DCs (Supplementary Figure 7). Thus, we propose that the effect of sCD83 on F-actin is independent of the MD-2/TLR4 pathway and that determination of the specific mechanism of sCD83 in DCs warrants further study.

Rab1a mediates ER–Golgi transport and some membrane molecules transport (Ali et al., 2004), and it also plays a role in autophagy (Webster et al., 2016). In addition, Rab1a regulates localization of members of the LRRK2-related kinase family to influence F-actin accumulation (Kicka et al., 2011), which further affects the accumulation of MHC-II at the site of DC-T contacts in our studying. Further, Rab1a regulates sorting of early endocytic vesicles involving Rab5, Rab7, Rab9, and Rab11, all of which would be required for MHC-II expression on DCs (Mukhopadhyay et al., 2014; Perez-Montesinos et al., 2017). Therefore, Rab1a may participate in antigen presentation processing by MHC-II. Further, Rab1a has a role in COPII vesicle traffic (Constantino-Jonapa et al., 2020; Westrate et al., 2020), which is important for MHC and antigen processing in the ER. Although Rab1a may have an important role in regulating antigen presentation processing by different means, a detailed understanding of the regulatory process of Rab1a in DCs warrants additional study.

Uveitis, an inflammatory disease involving the uvea, retina, retinal vessels, and/or vitreous body, can result in visual impairment and blindness (Rothova et al., 1996). The treatment of uveitis remains a challenging undertaking and an area of active research (Rothova et al., 1996; You et al., 2017; Zhao and Zhang, 2017). We demonstrated that increased sCD83 acts as a suppressive molecule in EAU alleviating symptoms of the disease by disrupting DC-T contact through IS inhibition in DCs, but not T cells (Supplementary Figure 3). This potential mechanism may underlie the decreased number of T cells and DCs following sCD83 treatment in mice with EAU and the amelioration of symptoms in affected animals. Furthermore, increased sCD83 in animals with EAU may be a result of self-regulation in the immune response, which warrants further investigation to better understand immune regulation in EAU. In addition, we found that administration of sCD83-treated DCs decreased the number of T cells and DCs in mice with EAU and reduced the symptoms of EAU. The mechanism of sCD83-treated DCs on EAU might be that these sCD83-treated DCs inhibit T cell activation by losing to interact with T cells well, or these sCD83-treated DCs would secrete IL-10 or IDO to inhibit T cell activation (Lin et al., 2018) or suppress the endogenous DCs by IL-10 to further inhibit T cell activation (Lin et al., 2018). The mechanism of sCD83-treated DC on EAU needs further study. Our findings may provide a new direction for the treatment of autoimmune uveitis using DCs.

To conclude, we demonstrate that sCD83 inhibits DC-T synapse formation by decreasing Rab1a activation to disrupt F-actin and MHC-II accumulation at sites of DC-T contact. Furthermore, our findings provide a possible mechanism of sCD83 on the role of DCs in IS and show that Rab1a may be a potential regulator of DC function. Finally, sCD83-treated DCs alleviate symptoms of EAU in mice, providing a potentially new avenue for EAU treatment.

## Data Availability Statement

The original contributions presented in the study are included in the article/Supplementary Materials, further inquiries can be directed to the corresponding author/s.

## Ethics Statement

The animal study was reviewed and approved by Shandong First Medical University & Shandong Academy of Medical Sciences.

## Author Contributions

WL designed this research, wrote the paper, and analyzed data. WL, SZ, and MF performed animal experiments, western blot, and flow cytometry experiment. QS, XL, and YY cultured cells and performed animal experiments. All authors agreed to the published version of the manuscript.

## Conflict of Interest

The authors declare that the research was conducted in the absence of any commercial or financial relationships that could be construed as a potential conflict of interest.
