# Supplementary material for: Soluble CD83 Regulates Dendritic Cell–T Cell Immunological Synapse Formation by Disrupting Rab1a-Mediated F-Actin Rearrangement
Source: Front Cell Dev Biol. 2021 Jan 22;8:605713. doi: 10.3389/fcell.2020.605713 (PMC7874230; doi:10.3389/fcell.2020.605713)
Supplement: Supplementary file 1 [file Data_Sheet_1.doc]

**Soluble CD83 regulates dendritic cell-T cell immunological synapse formation by disrupting Rab1a-mediated F-actin rearrangement**

Wei Lin^1^*, Shuping Zhou^1^, Meng Feng^1^, Yong Yu^1^, Qinghong Su^1^, Xiaofan Li^1^

1 Institute of Basic Medicine, Shandong Provincial Hospital Affiliated to Shandong First Medical University, Shandong First Medical University &Shandong Academy of Medical Science, Jinan, 250062, China

# **Correspondence:**

Wei Lin, Ph.D.

Institute of Basic Medicine, Shandong Provincial Hospital Affiliated to Shandong First Medical University, Shandong First Medical University &Shandong Academy of Medical Science

18877# Jingshi Road, Jinan, Shandong province, China, 250062,

Phone:(+86)-0531-82979938,

linw1978@163.com or weilin11@fudan.edu.cn

**Supplementary Figure Legend**

**
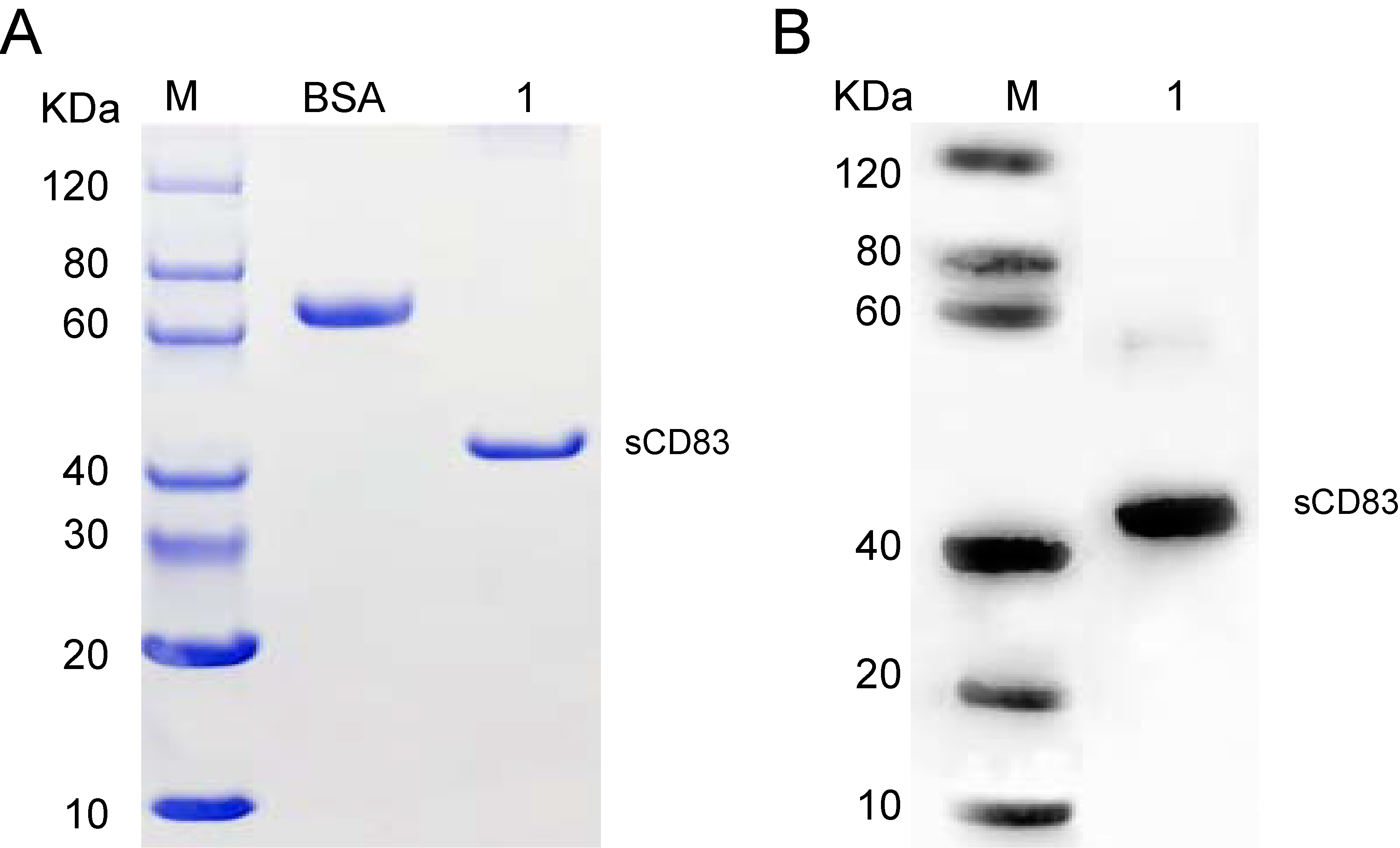
**

**FIGURE S1**│**The expression of sCD83 protein was detected by SDS-PAGE (A) and Western blot (B).** sCD83 were purified and detected by SDS-PAGE and Western blot, M: marker,1: sCD83 protein.

**
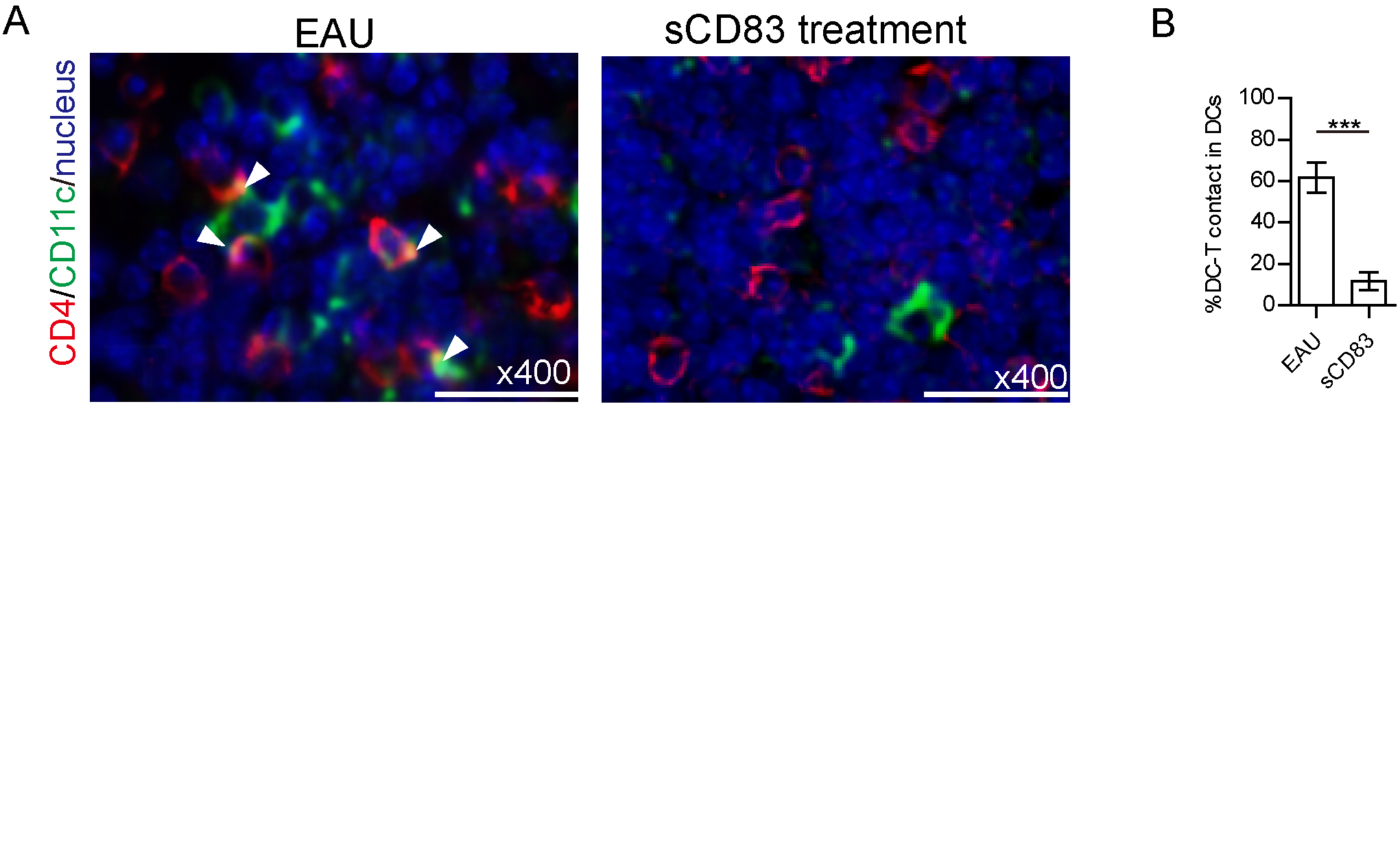
**

**FIGURE S2| The CD4^+^ T cells and CD11c^+^DC in the lymph nodes of EAU and sCD83 treated EAU mice. (A)** CD4^+^ T cells (red) and CD11c^+^ DC lymphocyte subpopulation (green) in the lymph nodes of EAU, and sCD83 treated EAU mice were detected by immunofluorescence. Nucleus is blue. Bar=20µm. (B) the percentage of DC-T contact were found in the lymph nodes of EAU and sCD83 treated EAU mice. Three visual fields/group, and twenty DCs/ field were selected to evaluate whether they can contact with T cells. Data are shown as mean±SEM. ***p< 0.001.

**
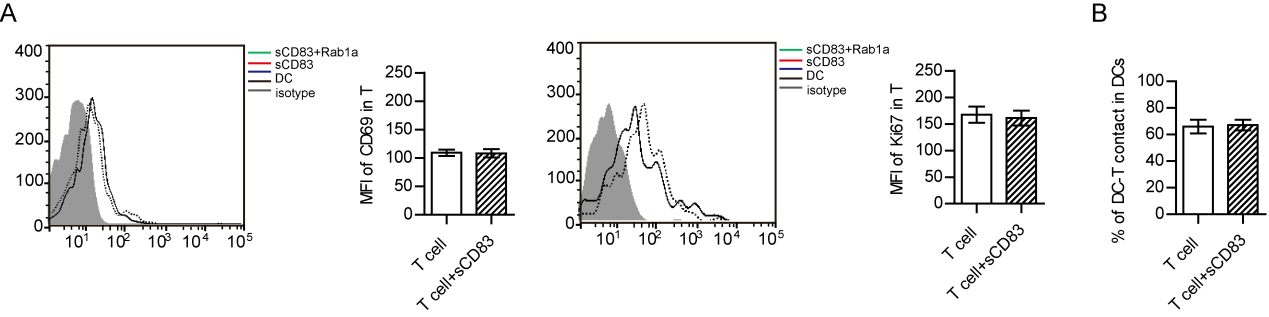
**

**FIGURE S3**│ **The effect of sCD83 on T cells.** (A) After sCD83 treated T cells, the expression of CD69 and Ki67 in T cells and (B) the percentage of DC-T contact in DCs were not significantly different from these without treatment. Data are from three separate experiments.


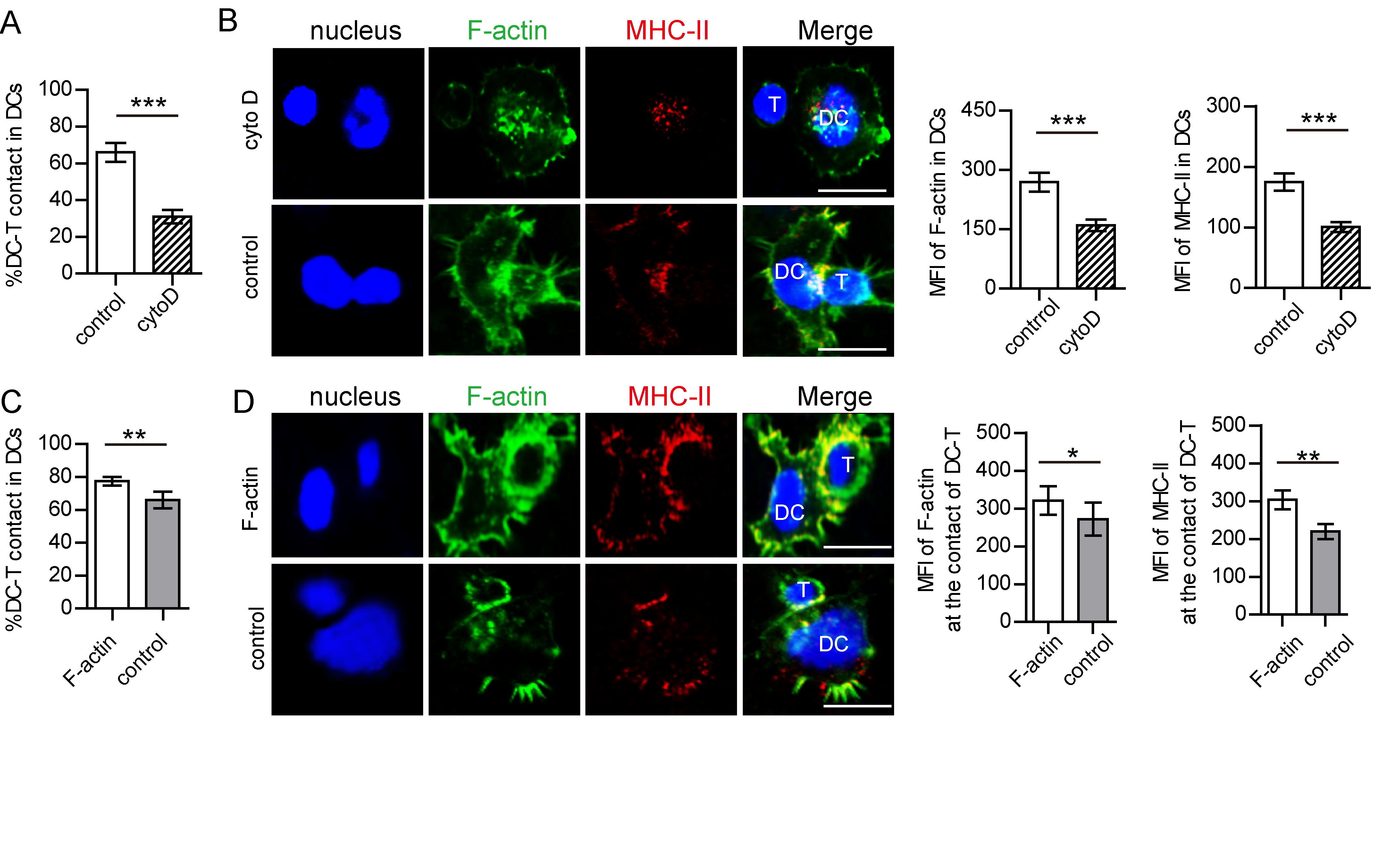


**FIGURE S4**│**The effect of cytochalasin D and F-actin on DC and T cell contact.** (A) The percentage of DC-T contact in cytochalasin D-treated-DCs and PBS-treated- DCs (control). (B) The distribution and expression of F-actin and MHC-II at cytochalasin D-treated-DC-T contacts and control group. (C) The percentage of DC-T contact in F-actin overexpressed DCs and empty plasmid-transfected DCs (control). (D) The distribution and expression of F-actin and MHC-II at F-actin overexpressed DCs-T contacts and control group. Bar=5μm. Nucleus is blue. Data of (A-D) are from three separate experiments and shown as mean±SEM. *p<0.05, **p <0.01, ***p< 0.001. Nucleus is blue. Bar=5μm.


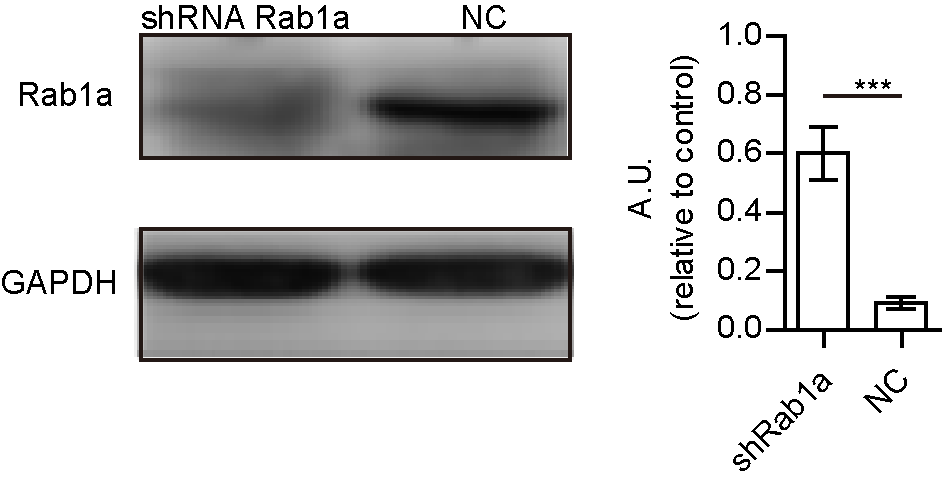


**FIGURE S5**│**The effect of shRNA-Rab1a on the expression of Rab1a in DC.** With shRNA-Rab1a or NC treatment, the expression of Rab1a were detected by western blot and analyzed by image J. Data are shown as mean±SEM. ***p< 0.001.


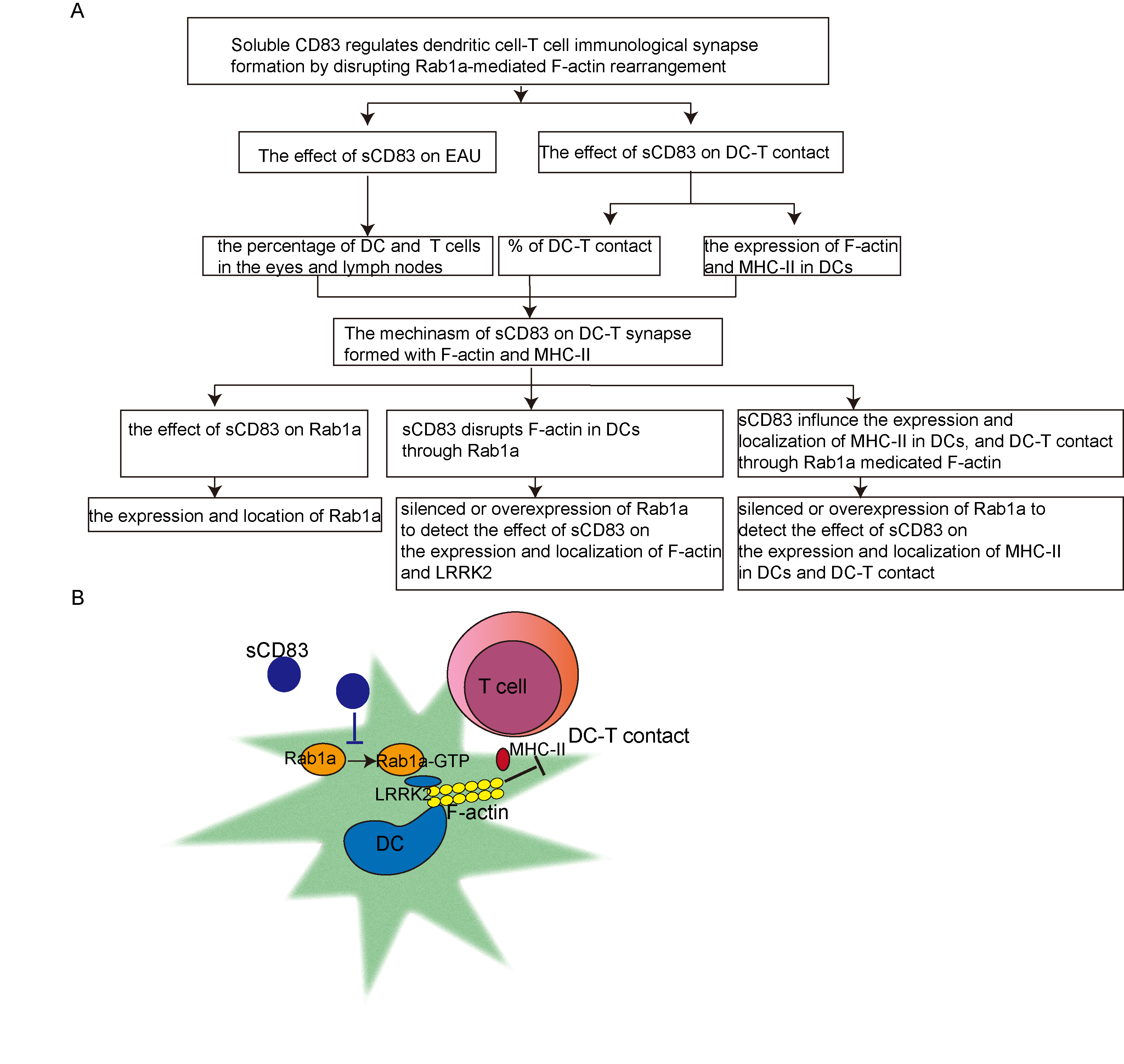


**FIGURE S6**│**The design and schematic diagram of this article.** (A) The roadmap of the experiment in this manuscript. (B) The model of sCD83 regulated DC-T synapse, which were formed with F-actin and MHC-II, by inhibiting Rab1a-GTP.


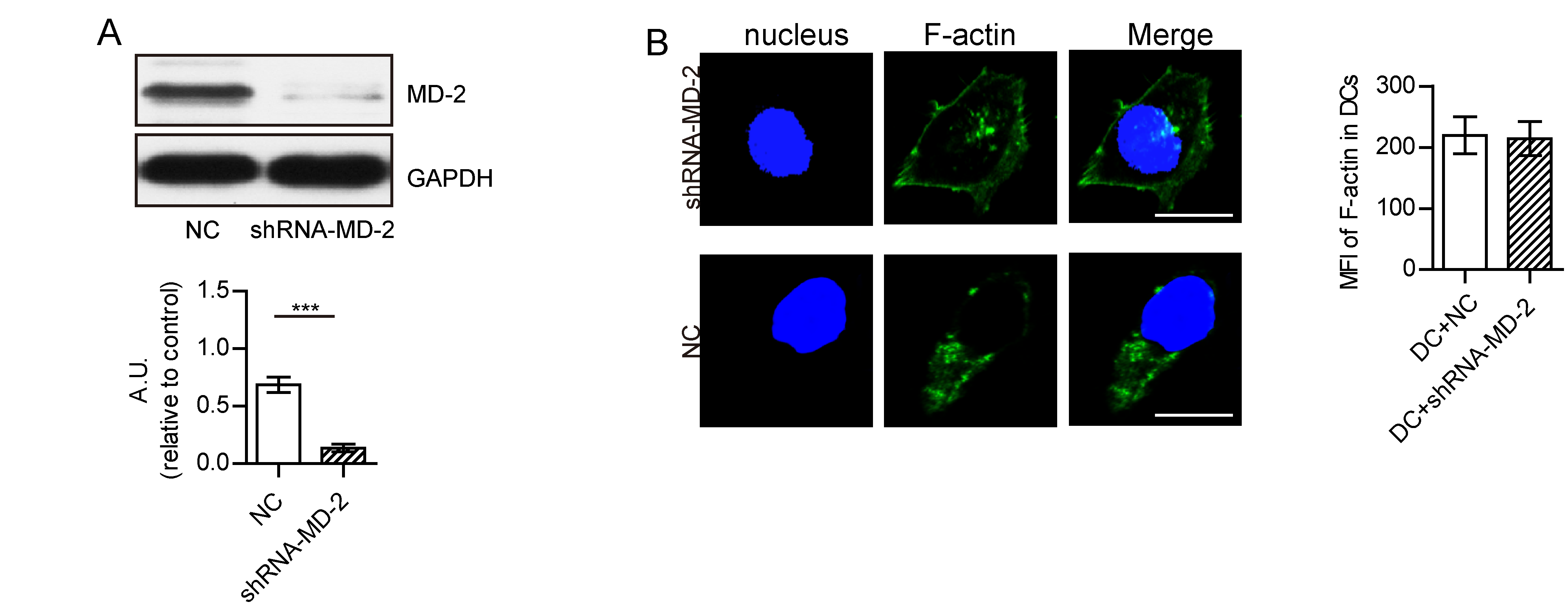


**FIGURE S7**│**The effect of MD-2 on F-actin arrangement in DC.** (A) With shRNA-MD-2 or NC treatment, the expression of MD-2 were detected by western blot, and (B) the distribution of F-actin (green) in DC2.4 cells were measured by confocal microscopy (left panel). The expression of F-actin in shRNA-MD-2 treated DC compared with NC treated DC (right panel). Bar=5μm. Nucleus is blue. Data are from three separate experiments. Data are shown as mean±SEM. ***p< 0.001.
